# Magnesium absorption as influenced by the rumen passage kinetics in lactating dairy cows fed modified levels of fibre and protein

**DOI:** 10.1017/S1751731118002963

**Published:** 2018-11-16

**Authors:** J.-L. Oberson, S. Probst, P. Schlegel

**Affiliations:** 1 Agroscope, Ruminant Research Unit, 1725 Posieux, Switzerland; 2 School of Agricultural, Forest and Food Sciences, Bern University of Applied Sciences, 3052 Zollikofen, Switzerland

**Keywords:** mineral, herbage, rumen volume, rumen passage rate, NDF

## Abstract

The potassium sensitive magnesium absorption through the rumen wall may be influenced by additional dietary properties, such as diet type, forage type or forage to concentrate ratio. These properties are likely associated to rumen passage kinetics modified by dietary fibre content. The study aimed to assess the effects of rumen passage kinetics on apparent Mg absorption and retention in lactating dairy cows fed modified levels of fibre. Six lactating Red-Holstein and Holstein cows, including four fitted with ruminal cannulas were randomly assigned to a 3 × 3 cross-over design. The experimental diets consisted of early harvested low NDF (341 g NDF/kg DM) and late harvested high NDF (572 g NDF/kg DM) grass silage (80% DM) and of concentrates (20% of DM). As the low-fibre diet was excessive in protein, a third high-fibre diet was formulated to be balanced in digestible protein with the low-fibre diet to avoid any eventual confounding effects of NDF and protein excess. All diets were formulated to contain iso-Ca, -P, -Mg, -K and -Na. Passage kinetics of solid and liquid phase of rumen digesta were evaluated using ruminal marker disappearance profiles. Cows fed the low-fibre diet had compared to the other diets, an up to 40% lower solid and 26% lower liquid phase volume of rumen digesta and a 10% numerically higher fractional rumen liquid passage rate. Rumen pH lost 0.6 units and Mg concentration in the rumen liquid phase tripled when cows were fed the low-fibre diet. Faecal Mg excretion was up to 14% higher in cows fed the low-fibre diet and Mg absorbability was 12% compared to up to 19% in other diets. Urinary Mg excretion in cows fed the low-fibre diet was half of the ones in the other treatments, but Mg retention was not affected. Dietary protein excess neither affected rumen passage kinetics nor Mg absorption and retention. Absorption of Mg was correlated with rumen liquid volume which both decreased with decreasing daily NDF intake (NDFi, 11.8 ± 2.4 l/kg NDFi). Consequently, daily Mg absorption decreased by 1.32 ± 0.28 g/kg decreasing NDFi. To conclude, in addition to the known antagonistic effect of dietary K, the present data indicate that Mg absorption was dependent from NDFi which modified rumen liquid volume, but was independent of dietary protein excess likely associated to low NDF herbages.

## Implications

Hypomagnesaemia remains an important topic in ruminant nutrition, especially in high yielding animals fed intensively produced herbage-based diets. Their potassium content is likely high and known to limit magnesium absorption. The provided data demonstrate that fibre intake is an additional antagonistic factor for magnesium absorption in dairy cows. Low-fibre intake reduces the rumen liquid volume, a possible reason for the observed limited magnesium absorption. Thus, in addition to dietary potassium, dietary fibre could be considered to formulate magnesium in dairy cattle diets.

## Introduction

The risk for hypomagnesaemia occurrence, clinically diagnosed as grass tetany in lactating cows (Sjollema, 1930) is increased with dietary characteristics of intensively managed herbages, such as high concentrations in K and rapidly fermentable protein and low concentrations in Mg (Meschy, 2010). Increasing dietary K linearly decreases Mg absorbability in cows (Adediji and Suttle, 1999; Weiss, 2004; Schonewille *et al.*, 2008), but the provided equations differ among authors resulting in relevant different Mg absorption rates for a given dietary K, especially when K is high. A K to diet type interaction (Suttle, 2010), a K to forage type interaction (Schonewille *et al.*, 2008) and a contrasting forage to concentrate (F:C) ratio (Adediji and Suttle, 1999; Schonewille *et al.*, 2002) were suggested as possible reasons for this lacking uniformity between equations. One common denominator of these suggestions is the modified rumen passage kinetics, namely rumen volumes (Vol) and passage rates (Kp). Modified diet type, forage type or F:C ratio influenced the solid phase Kp (Kp_S_, Lopes *et al.*, 2015), the solid phase Vol (Vol_S_, Stensig and Robinson, 1997; Kuoppala *et al.*, 2010), the liquid phase Kp (Kp_L_, Stensig and Robinson, 1997; Schonewille *et al.*, 2002) and the liquid phase Vol (Vol_L_, Stensig and Robinson, 1997; Schonewille *et al.*, 2002; Krämer *et al.*, 2013). As the rumen epithelium is the main absorption site for Mg in ruminants (Tomas and Potter, 1976), it was hypothesized that the modified rumen passage kinetics (Kp_S_, Vol_S_, Kp_L_ and Vol_L_) from herbage with modified fibre content would affect Mg absorption in lactating dairy cows. The protein excess in diets based on low fibre containing herbage may be associated to compromised Mg absorption as cows showed signs of tetany when fed intensively managed herbage (Kemp, 1960). As there is no clear evidence for a direct relation between protein excess and Mg absorption in cows (Weiss, 2004; Schonewille *et al.*, 2008, Martens *et al.*, 2018), especially under constant dietary K conditions, it was also hypothesized that Mg absorption would be independent from dietary protein excess associated to low-fibre herbage-based diets. If so, herbage fibre and its effect on rumen passage kinetics would be the explaining interaction for the various regressions associated to dietary K contents and this, independent from dietary protein.

## Material and methods

### Animals and experimental design

Six lactating Red-Holstein and Holstein cows (697 ± 61 kg BW, 130 ± 60 days in lactation, 5 ± 2 lactations, mean ± SD), including four with ruminal cannulas used for rumen kinetics measures, were selected from the Agroscope dairy herd (Posieux, Switzerland) and randomly assigned to a 3 × 3 cross-over design with three treatment diets and three consecutive periods. One period consisted of 14 days (first period) or 21 days (subsequent periods) adaptation in a tiestall followed by 7 days of collection in metabolic tiestall allowing hourly feed intake recordings (Balreader, Mettler Toledo, Greifensee, Switzerland) and total quantitative collection of faeces, urine and milk.

### Experimental diets

Herbages were mowed on the 1^st^ seasonal harvest when NDF concentration reached 350 (early harvest) and 550 g/kg DM (late harvest), respectively. The difference in NDF concentration aimed to be at least at 200 g/kg DM to expect modified rumen passage kinetics (Colucci *et al.*, 1990; Kuoppala *et al.*, 2010). The two cuts were pre-wilted, pressed in square bales without chopping and without additive, wrapped with plastic for fermentation and stored inside until their use. The silages originated from the same plot (660 m a.s.l., 46°7712’N, 07°1055’E) which consisted of 76% and 87% of graminea (Lolium perenne) and of 24% and 13% of clover (Trifolium pratense and repens) of the fresh matter weight in the early and late harvest, respectively.

Three experimental diets were formulated by using the two silages and three concentrates. Diet Fibre− consisted of the early harvest and of concentrate Fibre−. Diet Fibre+ consisted of the late harvest and of concentrate Fibre+ to obtain potentially modified rumen passage kinetics with diet Fibre−. The third diet (Fibre+CP) consisted of Fibre+ and of concentrate Fibre+CP to balance digestible protein concentration (PDIN) with diet Fibre−. The F:C ratio was set at 80:20 on DM basis. The relatively low proportion of concentrate is representative, under Swiss conditions, for dairy cows with such milk yields (Schmid and Lanz, 2013; Federal Office for Agriculture, 2017) and allowed to limit the dilution of NDF differences between Fibre− and Fibre+ or Fibre+CP considered as one major parameter to influence rumen Kp (National Research Council (NRC), 2001; Krizsan *et al.*, 2010). Based on the analysed nutrients of silage and ingredients to be included in the concentrates, the experimental diets were formulated to reach or exceed nutrient requirements (650 kg BW, 22 kg/day DM intake (DMI) and 30 kg/day milk yield) according to the Swiss feeding recommendations (Agroscope, 2015), except in Mg set at a marginal level of 2.3 g/kg DM. Diets were balanced in Ca, P, Mg, K and Na concentrations and in PDIN between Fibre− and Fibre+CP by using the three pelleted concentrates, produced on the on-site feed mill according to the formulation shown in Supplementary Table S1.

Cows were fed the silage *ad libitum* during the adaptation periods and fed according to their previous 7 days mean DMI during the collection periods. The concentrate was offered in a separate container placed on top of silage and its amount was fixed per cow on a weekly basis. Cows were offered their diets at 0730 and 1700 h and had *ad libitum* access to water.

### Marker preparation, labelling technique and data collection

Ytterbium-labelled fibre (Yb-NDF) and cobalt ethylenediaminetetraacetic acid (Co-EDTA) were used as marker for, respectively, the solid and liquid phase of rumen digesta. Fibre preparation and labelling technique (Supplementary Material S1) were adapted from Udén *et al*. (1980), Beauchemin and Buchanan-Smith (1989) and Ellis *et al*. (1994). Co-EDTA was prepared according to Udén *et al*. (1980).

Cows were weighted the day before and after each collection period. Intake of silage and concentrate was recorded as weight difference between offer and refusals on a daily basis. Water consumption was recorded daily during the collection period and after each rumen sampling. Samples of silages were collected daily, dried at 60° for 24 h and pooled per collection period. Samples of concentrates were collected daily and pooled per collection period. Individual diet refusals were collected at 0700 h, prior feeding, dried at 60° for 24 h and pooled per cow and collection period. At each milking (0600 and 1600 h), milk yields were determined and milk samples were taken. An aliquot of 0.7% (w/w) from each milking was pooled per cow and collection period and stored continuously at −20°C until analysis. Total faecal and urine excretion were collected and weighted daily at 0900 and 0930 h, respectively, during the collection period. The faeces were collected in a container placed beneath the metabolic tie stall. The urine was collected via urinals attached around the vulva with Velcro straps glued to the shaved skin into 50 l containers, whereas ∼2% of the 24 h collection was stabilized using 10% of sulfuric acid (2.5 mol/l). Daily homogenized aliquots of faeces (90 g, corresponding to ∼0.25% w/w) and urine (0.5% w/w) were pooled per cow and collection period and stored continuously at −20°C until analysis. Frozen faecal samples were lyophilized (Christ-Delta 1to 24 LSD; Martin Christ, freeze-drying technology GmbH, Osterode am Harz, Germany) for 72 h. Silage, concentrate and faecal samples were milled in 1 mm screen (Brabender mill, Brabender, Duisburg, Germany) and stored in sealed jars at room temperature until analysis.

On day 3 of the collection period, shortly before the morning meal, 200 g of Yb-NDF was introduced into the dorsal rumen, via the cannula, using a plastic tube (Ø8 × 70 cm). Subsequently, 50 g of Co-EDTA solubilized in 500 ml of water was introduced into the rumen via the cannula. Samples of rumen content were collected prior and 1, 2, 3, 5, 7, 10, 16 and 23 h after marker application. Sampling was timely enhanced compared to Schonewille *et al*. (2002) because of the expected slower Kp_S_ (NRC 2001). The rumen liquid phase was collected from the ventral rumen using a syringe equipped with a 40 cm long tube and a 2 mm pore size sieve. The pH was measured directly after collection (Consort P902; Consort bvba, Turnhout, Belgium). Rumen liquid samples were centrifuged at room temperature for 1 min at 1000 × g. The supernatant was centrifuged for 10 min at 12 000 × g and the resulting supernatant was stored continuously at −20°C until analysis. The rumen solid phase was collected per hand grabs taken in the dorsal, medial and ventral rumen. Each sample was rinsed six times under tap water using a sieve to remove eventual migrated solubilized Yb or Yb bound to micro-particles (Beauchemin and Buchanan-Smith, 1989). Rumen solid phase samples were dried at 60°C for 24 h, resulting in samples of 390 ± 14 g per cow, were ground using a 1 mm screen and stored in sealed jars at room temperature until analysis. Blood was sampled on day 5 of each collection period at 0800 h from the jugular vein using 9 ml Li-heparin vacutainer (Greiner Bio-One, Kremsmuenster, Austria). Samples were held in ice until centrifugation (15 min, 3000 × g) and the retrieved plasma was stored continuously at −20°C until analysis.

### Chemical analysis

Dry matter contents of silages, silage refusals, concentrates, rumen solid phase samples and faeces were determined thermogravimetrically by heating at 105°C for 3 h (Leco TGA 601, Mönchengladbach, Germany) and ash content was subsequently determined after incineration at 550°C until constant weight was attained. Crude protein content in silages, concentrates and faeces was calculated as 6.25 × nitrogen, where nitrogen was determined using the Dumas combustion method (AOAC procedure 990.03). Crude fibre content in silages and concentrates was determined after the samples were digested with successively H_2_SO_4_ and KOH, washed with acetone, dried at 130°C and finally ashed (VDLUFA method 6.1.4). Contents of ADF (VDLUFA method 6.5.2) and NDF (VDLUFA method 6.5.1) were analysed using the Fibretherm^®^ system (Fibretherm^®^ FT 12, C. Gerhardt GmbH&Co., Königswinter, Germany) and expressed inclusive residual ash. Crude fat content in silages and concentrates was determined as petrol ether extract after an acidic hydrolysis in boiling HCl for 1 h (VDLUFA method 5.1.1). Calcium, P, Mg, K and Na in dry ashed feedstuffs, Mg, Na and K in dry ashed faeces and rumen solid phase samples (h 5), Yb in the rumen solid phase samples and Co, Mg, K and Na in autoclaved (55 min at 235°C) rumen liquid samples (h 2, 5, 10) were analysed by inductively coupled plasma optical emission spectrometry (ICP-OES; Optima 7300 DV, Perkin Elmer, Schwerzenbach, Switzerland). Cobalt concentrations below 10 mg/kg were re-analysed by graphite furnace atomic absorption spectrometry (GF-AAS; Analyst 600 Perkin-Elmer, Perkin Elmer Corp., Norwalk, CT, USA). Blood plasma and urine samples were assayed using commercially available kits according to manufacturer’s instruction on a Lisa 200 autoanalyser (Biomérieux, Marcy l’Etoile, France) to determine Mg concentration, while K and Na were determined using ion selective electrodes method (Analyser Na^+^ K^+^ Ilyte^TM^; IGZ Instruments AG, Zürich, Switzerland). Milk samples were analysed for fat, protein, lactose and urea content using Fourier transformed IR spectrophotometry and for Mg, Na and K by GF-AAS. The laboratories were accredited with the certification ISO:170025.

### Calculation and statistical analysis

Data for rumen passage kinetic and mineral balances were analysed using R software (version 3.3.2; 2016-10-31). Cobalt concentrations in rumen liquid were logarithmically transformed and were subjected to a multiple linear regression using a mixed effect model (Bates *et al.*, 2016). Differences among treatment intercepts and slopes were tested using the Type III Wald *F* tests (Fox and Weisberg, 2011) and treatment effects were assessed with the *Posthoc* interaction analysis PHIA (Rosario-Martinez, 2015) using Tukey’s contrasts. Calculations for Vol_L_ and fractional and absolute Kp_L_ were conducted according to Schonewille *et al*. (1999) and are described along with Vol_S_ and fractional and absolute Kp_S_ in the Supplementary Material S2. Absolute Kp_L_ and Kp_S_ treatment effects were assessed using Friedman *χ* ^2^ test where animal (1 to 4) was set as block and diet (Fibre−, Fibre+CP and Fibre+) as factor.

Data for mineral balances were averaged per cow and collection period defined as experimental unit, and were subjected to ANOVA (Fox and Weisberg, 2011) following the model *Y*_*ijk*_ *= μ+Diet*_*i*_ *+ Period*_*j*_ *+ Animal*_*k*_ *+ ε*_*ijk*_, where *Y*_*ijk*_ is the response, *μ* the least-squares mean, *Diet*_*i*_ the fixed effect of the experimental diet (*i* = Fibre−, Fibre+CP, Fibre+), *Period*_*j*_ the fixed effect of the period (*j* = 1 to 3), *Animal*_*k*_ the random effect of the animal (*k* = 1 to 6) and *ε*_*ijk*_ the random error. Comparisons among means were calculated using the generalized linear hypothesis test GLHT (Hothorn *et al.*, 2008) using Tukey’s contrasts. Differences were considered as significant when *P* ⩽ 0.05 and trends were noted at *P* < 0.10.

## Results

### Diet and production parameters

Analysed and calculated nutrients in the consumed silages and concentrates are presented in Supplementary Table S2. The NDF concentrations in the two silages were sufficiently different (231 g NDF/kg DM) to expect modified rumen passage kinetics. Nutrient concentrations in the silages were according to expectations, except that K was slightly lower (2.2 g/kg DM) in the late silage than initially analysed for diet formulation. The nutrient contents and nutritive values of the effectively consumed experimental diets (Table 1) originate from the measured ingested quantities and the analysed contents of the sampled feedstuffs. The K and Mg concentrations were comparable among all consumed treatment diets, although they slightly differed by maximal 1.90 and 0.09 g/kg DM, respectively. The Fibre− and Fibre+CP diets were similar in PDIN as it only differed by 0.1 g/kg DM. The daily DMI of silage, concentrate and total diet did not differ (*P* > 0.10) among treatments (Table 2). The concentrate intake represented 21.0%, 20.5% and 20.2% of the total DMI in Fibre−, Fibre+CP and Fibre+, respectively, and was not different (*P* > 0.10) between each other. Water intake was reduced (*P* < 0.01) by 9.2% and 8.9% in cows fed Fibre+ compared to Fibre+CP and Fibre−, respectively. The faecal DM excretion was lower (*P* < 0.05) from cows fed Fibre− than Fibre+CP and Fibre+, whereas the faecal DM concentration (122 ± 4.5 g/kg) remained similar (*P* > 0.10) among treatments. The urinary excretion differed (*P* < 0.001) among treatments and the urinary urea concentration was higher (*P* < 0.001) when Fibre+CP (18.2 g/l) was fed compared to Fibre− (11.1 g/l) and Fibre+ (7.75 g/l). The daily produced energy-corrected milk was 8.5% lower (*P* < 0.05) in Fibre+ compared to Fibre+CP and Fibre−. Milk fat concentration was similar (*P* > 0.10) among diets and milk protein concentration was higher (*P* < 0.01) in cows fed Fibre− than Fibre+ and Fibre+CP. Milk urea concentration increased (*P* < 0.001) by up to 83% in cows fed Fibre+CP compared to the other diets.

**Table 1:** Nutrient composition of the consumed experimental diets fed to lactating dairy cows

Nutrients (g/kg DM)	Treatments^1^		
	Fibre−	Fibre+CP	Fibre+
Organic matter	914	918	919
CP	210	209	142
Crude fibre	149	265	266
NDF	297	472	483
ADF	163	288	291
Crude fat	50.4	34.9	28.5
Ash	85.8	81.9	81.5
Ca	6.61	6.63	6.73
P	3.82	4.20	4.07
Mg	2.32	2.34	2.41
K	27.6	26.3	25.7
Na	1.70	1.53	1.56
NE_L_ (MJ)	7.32	5.66	5.70
PDIE	92	103	94
PDIN	132	132	94

NE_L_ *=* net energy for lactation. Protein digestible in the small intestine (Vérite *et al.*, 1979) calculated from its non-degradable N and degradable N contents (PDIN) or its rumen available energy content (PDIE).

1 Treatments consisted of 80% (DM basis) of early (Fibre−) or late (Fibre+CP; Fibre+) harvested grass silage and 20% of their respective concentrate.

**Table 2:** Dietary component intake and product excretion in lactating cows (*n* = 6 per treatment) fed the experimental diets

Parameters	Treatments^1^			SEM	*P*-value
	Fibre−	Fibre+CP	Fibre+		
Intake (kg DM/day)					
Grass silage	15.2	14.7	14.3	0.43	0.23
Concentrate	3.98	3.80	3.61	0.125	0.14
Total	19.2	18.4	17.9	0.52	0.13
Organic matter	17.5	17.1	16.5	0.55	0.43
CP	4.04^a^	3.89^a^	2.56^b^	0.198	<0.001
NDF	5.70^b^	8.78^a^	8.67^a^	0.434	<0.001
Water (l/day)	90.4^a^	97.8^a^	82.4^b^	5.34	0.004
Excretion (kg/day)					
Faeces^2^ (DM)	4.21^b^	4.96^a^	5.07^a^	0.197	0.04
Urine	36.2^a^	31.6^b^	24.9^c^	1.39	<0.001
Energy-corrected milk	27.2^a^	26.9^a^	24.8^b^	1.12	0.02
Milk fat (%)	4.24	4.32	4.39	0.123	0.74
Milk protein (%)	3.37^a^	3.25^b^	3.23^b^	0.062	0.002
Milk urea (mg/dl)	26.7^b^	43.1^a^	23.5^b^	2.26	<0.001
Digestibility (% of intake)					
Organic matter	79.2^a^	74.5^b^	73.4^b^	0.72	<0.001
CP	67.8^b^	75.6^a^	64.9^b^	1.24	<0.001
NDF	79.0^a^	72.4^b^	70.0^b^	1.05	<0.001

a,b,c Values within a row with different superscripts differ significantly at *P* < 0.05.

1 Treatments consisted of 80% (DM basis) of early (Fibre−) or late (Fibre+CP; Fibre+) harvested grass silage and 20% of their respective concentrate.

2 *P*-value given by non-parametric Friedman’s *χ* ^2^ test.

### Rumen volume, passage rate and mineral contents

The Vol_L_ decreased by up to 26% (*P* < 0.05) when cows were fed Fibre−, but the fractional Kp_L_ was not affected (*P* > 0.10) by the diets although it was numerically increased by 10% when cows were fed Fibre− (Table 3). The resulting absolute Kp_L_ was slower (*P* < 0.05) in cows fed Fibre− than Fibre+CP and Fibre+. Similar to Vol_**L**_, Vol_S_ decreased (*P* < 0.001) by up to 40% when cows were fed Fibre−, but fractional Kp_S_ was not affected (*P* > 0.10) by the diets. Finally, absolute Kp_S_ resulted in similar differences (*P* < 0.05) among diets as observed for volume. The Vol_**L**_ and Vol_**S**_ were correlated with their respective absolute Kp (liquid: Pearson’s *r* = 0.68, *df =* 10, *P* = 0.02; solid: Pearson’s *r* = 0.88, *df =* 10, *P* < 0.001), but both fractional Kp were independent (*P* > 0.10) from other parameters. No correlation was found between the ruminal liquid and solid phase parameters, except for their respective volume (Pearson’s *r* = 0.63, *df =* 10, *P* = 0.03).

**Table 3:** pH, mineral concentrations and passage kinetics of the liquid and solid phase of rumen digesta in lactating cows (fistulated; *n* = 4 per treatment) fed the experimental diets

Parameters	Treatments^1^			SEM	*P*-value
	Fibre−	Fibre+CP	Fibre+		
Liquid phase volume (l)	116^b^	146^a^	156^a^	7.2	0.02
Passage rate					
Fractional^2^ (%/h)	17.5	16.0	15.9	0.65	0.11
Absolute^3^ (l/h)	19.4^b^	23.3^a^	24.7^a^	1.10	0.04
Solid phase volume (kg NDF)	3.69^b^	5.72^a^	6.20^a^	0.790	<0.001
Passage rate					
Fractional (%/h)	1.50	1.57	1.46	0.030	0.32
Absolute^3^ (g/h)	55.0^b^	88.0^a^	87.1^a^	0.33	0.05
Rumen pH	5.84^b^	6.50^a^	6.46^a^	0.034	<0.001
Rumen liquid phase					
K (mmol/l)	34.8	32.5	28.9	0.88	0.07
Mg (mmol/l)	3.06^a^	0.60^b^	0.93^b^	0.021	<0.001
Na (mmol/l)	78.9^b^	90.1^a^	91.3^a^	1.89	0.01
Rumen solid phase					
K (g/kg DM)	0.78	1.14	1.45	0.181	0.60
Mg (g/kg DM)	0.72	0.73	0.68	0.032	0.89

a,b Values within a row with different superscripts differ significantly at *P* < 0.05.

1 Treatments consisted of 80% (DM basis) of early (Fibre−) or late (Fibre+CP; Fibre+) harvested grass silage and 20% of their respective concentrate.

2 The model included a negative quadratic effect of time. Values represent the maximal slopes.

3 Values represent the maximal slopes. *P*-value given by non-parametric Friedman’s *χ* ^2^ test.

The Mg and K concentrations in the solid phase of rumen digesta were similar between diets (*P* > 0.10, Table 3). In the liquid phase of rumen digesta, the concentration of K tended to be 20% higher (*P* < 0.10) in cows fed Fibre− compared to Fibre+ and the concentrations of Mg tripled (*P* < 0.001), Na was 13% lower (*P* < 0.05) and ruminal pH was up to 0.66 units lower (*P* < 0.001) in cows fed Fibre− compared to Fibre+CP and Fibre+.

### Nutrients digestibility and mineral balance

Diet Fibre− resulted in higher organic matter (OM) and NDF digestibility (*P* < 0.001; Table 2) and Fibre+CP resulted in higher CP digestibility (*P* < 0.001). Daily intake of Ca (124 ± 4.0 g/day), P (74.8 ± 2.5 g/day) and Mg were similar (*P* > 0.10) among diets, but daily intake of K (*P* < 0.01) and Na (*P* < 0.001) were slightly higher when Fibre− was fed compared to Fibre+CP and Fibre+. Faecal Mg excretion was up to 4.2 g/day higher (*P* < 0.01) in cows fed Fibre− than Fibre+CP and Fibre+ (Table 4). Apparent Mg absorption was numerically (*P* > 0.10) reduced and apparent Mg absorbability tended (*P* < 0.10) to be reduced in cows fed Fibre− by, respectively, up to 3.1 g/day and 7% units compared to Fibre+CP and Fibre+. In contrast with all other cows, one individual had the highest Mg absorption and absorbability when fed Fibre−. The reason remains unclear, but this cow had the lowest faecal DM excretion and the highest OM digestibility. The data removal from that individual resulted in lower (*P* < 0.05) Mg absorption and absorbability for Fibre− compared to Fibre+.

**Table 4:** Balance of Mg and K in lactating cows (*n* = 6 per treatment) fed the experimental diets

Parameters	Treatments^1^			SEM	*P*-value
	Fibre−	Fibre+CP	Fibre+		
Magnesium (g/day)					
Intake	44.6	43.0	43.2	1.21	0.60
Faecal excretion	39.2^a^	35.8^b^	35.0^b^	1.28	0.007
Apparent absorption	5.24	7.60	8.30	0.584	0.12
% of intake	11.9	17.5	18.9	1.37	0.08
Urinary excretion	1.74^b^	3.31^a^	3.47^a^	0.249	<0.001
Milk excretion	2.85^ab^	2.94^a^	2.53^b^	0.140	0.02
Retention	0.59	1.33	2.26	0.388	0.56
% of intake	1.26	3.04	4.86	0.924	0.61
Potassium (g/day)					
Intake	536^a^	487^ab^	461^b^	16.0	0.01
Faecal excretion	32.6^b^	47.0^a^	40.6^ab^	3.67	0.02
Apparent absorption	500^a^	439^b^	421^b^	13.5	<0.001
% of intake	94.0^a^	90.6^b^	91.4^b^	0.62	<0.001
Urinary excretion	477^a^	416^b^	403^b^	13.0	<0.001
Milk excretion	43.3^a^	41.2^ab^	37.1^b^	2.64	0.01
Retention	−20.9	−17.9	−19.4	2.87	0.95
% of intake	−3.90	−3.57	−4.57	0.915	0.88
Magnesium per K					
Apparent absorption (mg/g)	10.5^b^	17.4^a^	19.5^a^	1.45	0.01

a,b Values within a row with different superscripts differ significantly at *P* < 0.05.

1 Treatments consisted of 80% (DM basis) of early (Fibre−) or late (Fibre+CP; Fibre+) harvested grass silage and 20% of their respective concentrate.

Although, the amount of daily urine excretion was higher (*P* < 0.001) in cows fed Fibre−, the urinary Mg concentration was half and one-third as high as in cows fed Fibre+CP and Fibre+, respectively (2.07, 4.38 and 5.88 mmol/l, respectively, *P* < 0.001), resulting in 49% lower (*P* < 0.001) urinary Mg excretion in cows fed Fibre− compared to Fibre+CP and Fibre+. Milk Mg excretion from cows fed Fibre+ was lower (*P* < 0.05) than Fibre+CP although milk Mg concentration was similar (*P* > 0.10) among treatments. The Mg retention was positive (*P* < 0.05) and similar (*P* > 0.10) among diets. Blood plasma Mg tended (*P* < 0.10) to be lower in cows fed Fibre− (0.87 mmol/l) than Fibre+ (0.97 mmol/l) and Fibre+CP (1.02 mmol/l). Although K intake was higher (*P* < 0.01) in cows fed Fibre− compared to Fibre+, their faecal K excretion was lower (*P* < 0.05) than those fed Fibre+CP diet. Apparent K absorbability was relatively high (91% to 94%) and higher (*P* < 0.001) in cows fed Fibre− than Fibre+CP and Fibre+. Urinary and milk K excretions were also higher (*P* < 0.001 and *P* < 0.05, respectively) in cows fed Fibre− than Fibre+CP and Fibre+, respectively, resulting in a slight negative (*P* < 0.001) K retention, which was similar (*P* > 0.10) among treatments. Per gram K absorbed, the absorbed Mg declined (*P* < 0.01) by up to 46% in cows fed Fibre− compared to Fibre+CP and Fibre+. The Na absorbability was higher (*P* < 0.001) for Fibre− (85.4%) than Fibre+CP (76.3%) and Fibre+ (72.5%) and the Na retention was also negative and comparable (data not shown; *P* > 0.10) among treatments.

## Discussion

### Rumen passage rate and volume

**Figure 1:**
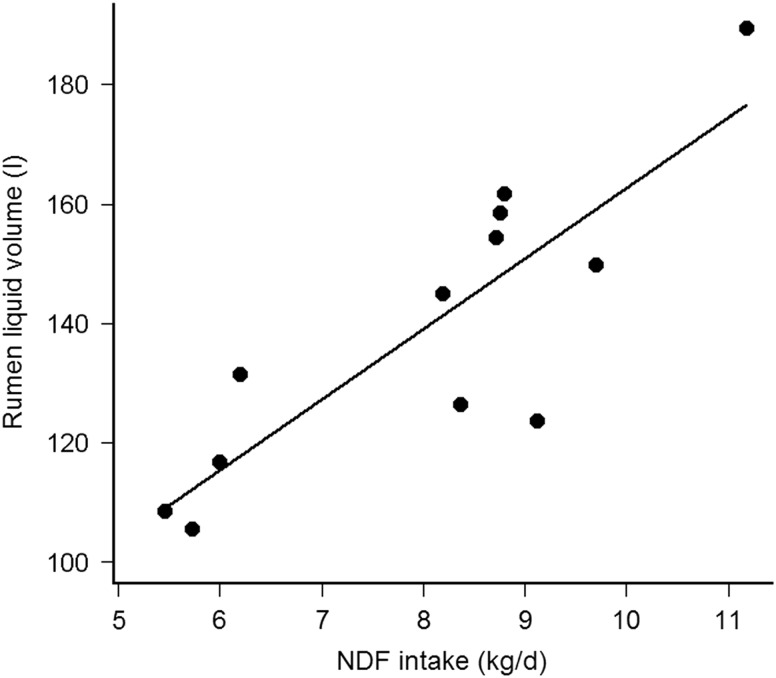
Relationship between rumen liquid phase volume (Vol_L_, l) and daily NDF intake (kg/day) in cows (fistulated; *n* = 4 per treatment).

The Kp_S_ was comparable with the Kp of cell walls estimated by rumen evacuation (Huhtanen *et al.*, 2007) and with the model correcting for forage type (Krizsan *et al.*, 2010), but less with the model using DMI as percentage of BW, forage NDF and F:C ratio (NRC, 2001). The diet independent fractional Kp_S_ support the findings of Kuoppala *et al*. (2010) who observed that Kp_S_ of indigestible NDF was not affected between early and late harvested grass silage. With comparable dietary NDF, Vol_S_ from Fibre+CP and Fibre+ were similar to the NDF pool estimated by rumen evacuation method (Kuoppala *et al.*, 2010). Although Krämer *et al*. (2013) observed similar Vol_S_, between maize and grass silage-based diets, the difference in Vol_S_ between Fibre− and Fibre+CP or Fibre+ are concordant with Stensig and Robinson (1997) and Kuoppala *et al*. (2010) who observed increasing pools of DM, OM, NDF and indigestible NDF, by feeding grass silages with increasing fibre content. Thus, the limited NDF intake (NDFi) reduces the physical fill of the rumen, but not the Kp_S_ (Kuoppala *et al.*, 2010). The fractional Kp_L_ was within the range issued from lactating cows fed grass silage based diets (Krämer *et al.*, 2013) and presenting similar DMI (Colucci *et al.*, 1990). The constant F:C ratio and the similar DMI between treatment diets, known to influence Kp_L_ (Stensig and Robinson, 1997; Schonewille *et al.*, 2002), allow the numerical differences in Kp_L_ to be attributed to the silages mainly differing in their NDF concentration. Nevertheless, the daily water consumption might have contributed to explain part of the Kp_L_ variability because they were correlated (Pearson’s *r* = 0.64, *df =* 10, *P* = 0.03). The Vol_L_ was higher than found in studies with comparable milk yield or similar forages (Kuoppala *et al.*, 2010; Krämer *et al.*, 2013), which can be explained by the up to 200 kg heavier cows and the higher F:C ratio in the present study. Reported as % of BW, Vol_L_ was 16% to 21% of BW in the present study and 12% to 18% of BW according to Krämer *et al*. (2013). The dietary NDF concentration was however higher (484 *v*. 378 g/kg DM) in the present study than in Krämer *et al*. (2013). Reported per kilogram BW and at similar dietary NDF, the present Vol_L_ are thus consistent with other studies. Increasing the F:C ratio (Colucci *et al.*, 1990; Stensig and Robinson, 1997) or feeding a grass compared to a maize silage-based diet (Krämer *et al.*, 2013) increased the Vol_L_. Here, the constant F:C ratio and the similar DMI between treatments allow the observed differences in Vol_L_ to be attributed to the silages mainly differing in their NDF concentration. Furthermore, Vol_L_ was not related to the daily water intake (Pearson’s *r* = −0.12, *df =* 10, *P* = 0.72), but was highly correlated with the daily NDFi (Pearson’s *r* = 0.85, *df =* 10, *P* < 0.001), which support previous data (Stensig and Robinson, 1997; Krämer *et al.*, 2013). Adjusted by cow, Vol_L_ increased by 11.8 ± 2.4 l per kg daily NDFi (*R* ^2^_marginal_ = 0.70, *P* < 0.001, Figure 1). Absolute Kp_L_ was affected by dietary treatments, because it was correlated with Vol_L_ but not with fractional Kp_L_. Absolute Kp_L_ may thus be less appropriate than Vol_L_ and fractional Kp_L_ to evaluate rumen kinetics. Finally, the rumen kinetics were considered as sufficiently modified between diet Fibre− and the other diets to study the Mg balance according to the defined hypothesis.

### Mineral balance, rumen mineral contents and their relation to rumen passage kinetics

According to the regression slopes to estimate Mg absorbability related to dietary K (Adediji and Suttle, 1999; Weiss, 2004; Schonewille *et al.*, 2008; Meschy, 2010), the 27 g K/kg DM from the present study, result to a Mg absorbability between 12% and 22%. The lower range is attributed to Adediji and Suttle (1999) based on diets including concentrates and to Weiss (2004) using maize silage-based diets. The higher range is attributed to Adediji and Suttle (1999) based on diets without concentrates and to Schonewille *et al*. (2008) using herbage-based diets without concentrates. This wide range of Mg absorbability was covered by the cows fed the experimental diets formulated to be iso-K. The lower than initially analysed K concentration in the consumed late harvested silage and its numerically lower intake explain the slight lower dietary K concentration (1.9 g/kg DM) and the reduced daily K intake of cows fed Fibre+ than Fibre−. This difference leads, according to the existing regressions for Mg absorbability (Adediji and Suttle, 1999; Weiss, 2004; Schonewille *et al.*, 2008; Meschy, 2010) and Mg absorption (Schonewille *et al.*, 2008) to a theoretical modification for diet Fibre− of, respectively, −0.6% to −1.0% units and +0.11 g/day. Absorption is improved as the proposed equation includes Mg intake. The potential impact on Mg absorbability of the slight difference in K content and intake between the experimental diets can thus be considered as irrelevant in relation to the modified 7% units between Fibre− and Fibre+. In addition, it has been suggested that the depressing effect of dietary K on Mg absorption may be attenuated with high dietary K levels (Martens *et al.*, 2018) such as contained in the present study. The impaired Mg absorption in cows fed diet Fibre− lead to a compensated lower urinary Mg excretion with a concentration below the considered as normal physiological range of 5 to 10 mmol/l (Meschy, 2010). This homeostatic counter regulation capacity started to be limited as plasma Mg level tended to be lower in cows fed Fibre−.

**Figure 2:**
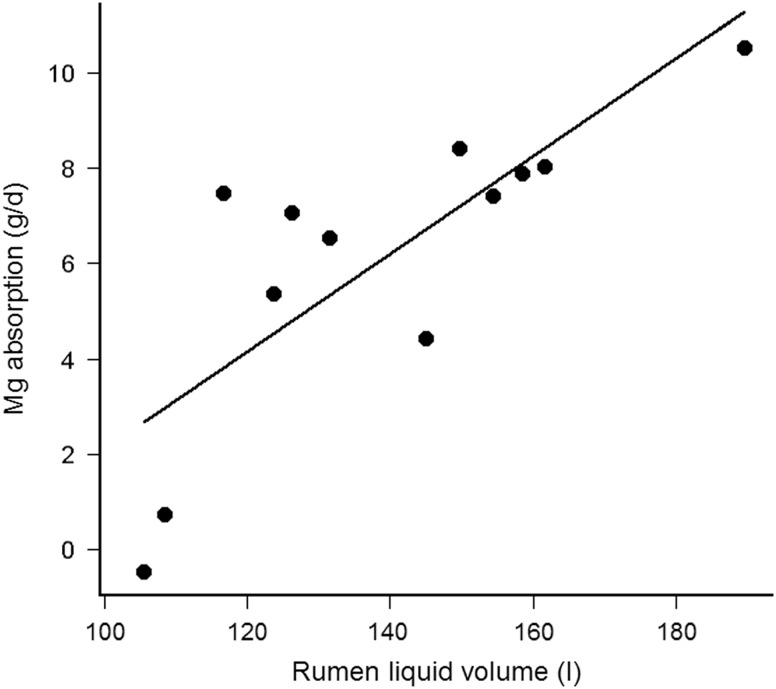
Relationship between rumen liquid phase volume (Vol_L_, l) and apparent Mg absorption (g/day) in cows (fistulated; *n* = 4 per treatment).

**Figure 3:**
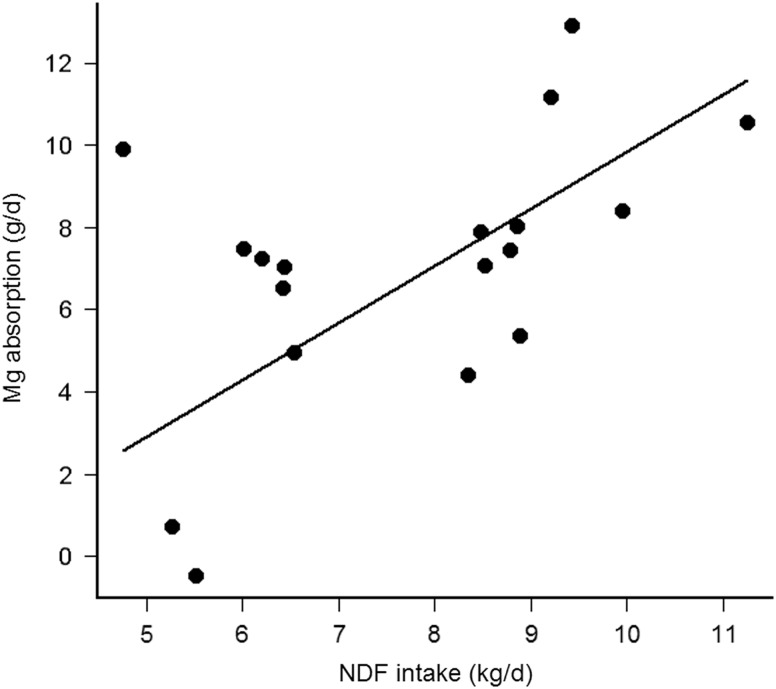
Relationship between apparent Mg absorption (g/day) and daily NDF intake (kg/day) in cows (*n* = 6 per treatment).

Magnesium absorption was positively correlated with Vol_L_ (Pearson’s *r* = 0.79, *df* = 10, *P* = 0.002), but not with fractional Kp_L_ (Pearson’s *r* = −0.45, *df =* 10, *P* = 0.14). Furthermore, Vol_L_ (*P* = 0.001), rather than Kp_L_ (*P* = 0.82), pH (*P* = 0.92) or K in the rumen liquid phase (*P* = 0.56) contributed to the explained variance in Mg absorption. Finally, Vol_L_ was not correlated with K (Pearson’s *r* = −0.26, *df =* 10, *P* = 0.80), but was negatively correlated (Pearson’s *r* = −2.85, *df =* 10, *P* = 0.02) with Mg in the rumen liquid phase. Magnesium absorption was thus solely dependent from Vol_L_ and decreased by 0.10 ± 0.02 g/day per l decrease of Vol_L_ (*R* ^2^_marginal_ = 0.60, *P* < 0.001, Figure 2). Finally, the Mg absorption was positively correlated with NDFi (Pearson’s *r* = 0.54, *df =* 16, *P* = 0.02, without the interacting cow (upper point left) *r* = 0.70, *P* = 0.002) and the Mg absorption decreased by 1.32 ± 0.28 g/day per kg decrease of NDFi (*R* ^2^_marginal_ = 0.48, *P* < 0.001, Figure 3). The present study thus demonstrates that the NDF dependent rumen passage kinetics, especially represented by Vol_L_, could be the explanatory variable for modified Mg absorbability, so far, hypothesized as influence of diet type (Suttle, 2010), forage type (Schonewille *et al.*, 2008) or contrasting F:C ratio (Adediji and Suttle, 1999; Schonewille *et al.*, 2002).

Further investigations are certainly necessary to explain the effect of NDFi on Mg absorption at rumen level. In the present study, the highest Mg concentration in the rumen liquid phase was in cows fed Fibre−. This phenomenon is explained by the reduced Vol_L_ and possibly also by an increased Mg solubilization as, according to Table 3, Mg solubility was 49%, 20% and 29% in cows fed diet Fibre− and diets Fibre+CP and Fibre+, respectively. The ruminal pH was also lowest in cows fed diet Fibre−, which may have favoured the increased Mg solubility. An increased Mg solubility is favourable for Mg absorbability (Schonewille *et al.*, 2002; Schonewille, 2013; Martens *et al.*, 2018). However, the potential benefits of a lower ruminal pH and a higher Mg solubility finally compromised Mg absorption in cows fed Fibre−. The lower Vol_L_ or rumen fill in cows fed diet Fibre− may have led to a physically limited contact surface between the rumen wall and the liquid phase and impaired Mg absorption, although the Mg concentrations in the rumen liquid phase were much lower than the ones necessary to saturate ruminal Mg absorption *in vivo* (Jittakhot *et al.*, 2004). The observed consistency of the ruminal content was less structured and the rumen liquid phase was much more viscous when cows were fed diet Fibre− in comparison with the other diets. It is open if such properties impact the physical contact of nutrients with rumen wall or impact the Mg ionization, necessary for rumen epithelial transport of Mg^2+^ (Martens *et al.*, 2018).

### Protein excess

When PDIN was fed excessively the resulted increased milk and urinary urea was expected. However, there was no influence on Mg absorption and retention over a period of 3 weeks. These results are inconsistent with Care *et al*. (1984) and Martens *et al*. (1988) who showed a reduced Mg absorption by infusing ammonium chloride into the rumen, but are concordant with studies on lactating dairy cows (Weiss, 2004) and sheep (Gäbel and Martens, 1986). According to Gäbel and Martens (1986), a dietary protein excess may depress Mg absorbability during a few days, which is recovered by adaptation of the rumen epithelium. Thus, a progressive switch to an excess of CP or PDIN as it occurs when changing, for example, from a conserved maize-herbage-based diet to pasture in springtime, an eventual hypomagnesaemia is not to be attributed to dietary protein excess.

## Conclusion

As NDFi was, independent from dietary K and protein, linearly related with the rumen liquid volume and with Mg absorption, the NDFi may be in addition to the known antagonistic effect of dietary K, the explanatory variable for modified Mg absorbability, so far, hypothesized as influence of diet type, forage type or contrasting F:C ratio. Further research is required to possibly implement dietary fibre into dietary K to Mg absorbability regressions.

## Supplementary material

For supplementary material accompanying this paper visit http://dx.doi.org/10.1017/S1751731118002963. click here to view supplementary material
