# Supplementary material for: Magnesium absorption as influenced by the rumen passage kinetics in lactating dairy cows fed modified levels of fibre and protein
Source: Animal. 2018 Nov 16;13(7):1412–20. doi: 10.1017/S1751731118002963 (PMC6581963; doi:10.1017/S1751731118002963)
Supplement: Supplementary file 1 [file S1751731118002963sup001.docx]

Magnesium absorption as influenced by the rumen passage kinetics in lactating dairy cows fed modified levels of fibre and protein (*animal* journal)

J.-L. Oberson^1,2^, S. Probst^2^ and P. Schlegel^1^

^1^ Agroscope, Ruminant Research Unit, 1725 Posieux, Switzerland

^2^ School of Agricultural, Forest and Food Sciences, Bern University of Applied Sciences, 3052 Zollikofen, Switzerland

Supplementary material S1

Marker preparation and labelling technique

Solid Phase Marker. Three batches of 1.2 kg dried and chopped (1-4 cm length) high NDF grass silage were added into a NDF-solution (20 l/kg, NDF Lösung, Zeller Gmbh, Hohenems, Austria), heated at 60°C for at least 24h and brewed manually twice during this laps. The fibres were rinsed 6 times under tap water using a 3 mm sieve to remove the detergent and then dried at 60°C for 24h. Ytterbium-NDF was prepared by soaking 1 kg of fibre (95% DM) in 15 l demineralized water containing 50 g of YbCl_3_ (Sigma-Aldrich Chemie GmbH, Buchs, Switzerland) for 24h, to obtain 29.5g Yb per kg fibre DM.

The labelled fibres were rinsed 6 times under tap water using a 3 mm sieve to remove unbound material and small particles and then soaked overnight in a solution of demineralized water adjusted to pH 4 with acetic acid as proposed by Ellis et al. (1994) to remove residual loosely bound marker. The labelled fibres were then rinsed again under tap water, dried at 60°C for 24h and stored in plastic bags at room temperature until use. The three batches of labelled fibres contained 15.5 ± 1.1 mg Yb/g DM or 19.8 ± 1.04 mg Yb/g of NDF.

Supplementary material S2

Calculation of the rumen passage kinetic parameters

Liquid phase parameters. Cobalt concentration at time h 0 was estimated per cow and collection period using the intercept of the model. The rumen liquid phase volume (Vol_L_, l) was calculated by dividing the infused Co with the Co concentration at time h 0. The fractional passage rate of the rumen liquid phase (Kp_L_, %/h) represented the slope of the log-linear regression and was modelled using a quadratic time effect. The absolute Kp_L_ (l/h) equalled the fractional Kp_L_ multiplied by Vol_L_.

Solid phase parameters. The fractional (%/h) and absolute (kg/h) passage rate of the rumen solid phase (Kp_S_) and the rumen solid phase volume (Vol_S_) were estimated as for the liquid phase parameters, using Yb-NDF. Ytterbium concentrations from the first three hours after marker application (*Yb_t(h=1-3)_*) were calculated per cow and period due to their heterogeneity using *Yb_t(h)_ = (D/DMI_t(h)_)*0.75*e^^((-Kp*t(h))^* including the marker dosage (D) at application, the dry matter intake (DMI) at time t_(_*_h_*_)_ (*DMI_t(h)_)*, the proportion (0.75) of the raft rumination pool potentially mixing the marker in Vol_S_ (adapted from Ellis *et al*., 1994) and the estimated individual fractional Kp according to NRC (2001). Logarithmically transformed X: ln(t_(_*_h_*_=1-23)_) and Y: ln(*Yb_t(h)_*) axes resulted in a linear relationship.

Supplementary Table S1 *Ingredients of the experimental concentrates fed to dairy cows*

| Ingredients (g/kg) | Concentrates^1^ | | |
| --- | --- | --- | --- |
|  | Fibre- | Fibre+CP | Fibre+ |
| Wheat grains | 245 | 245 | 245 |
| Barley grains | 292 |  | 292 |
| Maize grains | 406 |  | 130 |
| Maize gluten meal 60%CP |  | 245 | 245 |
| Soybean meal 48%CP |  | 337 |  |
| Urea |  | 58.4 |  |
| Beet molasses | 20.0 | 20.0 | 20.0 |
| Fat tallow mixed |  | 35.8 |  |
| Calcium carbonate | 12.4 | 20.5 | 20.6 |
| Magnesium oxide | 6.70 | 7.20 | 8.40 |
| Monocalcium phosphate |  | 13.7 | 16.0 |
| Iodized salt | 15.4 | 15.4 | 15.4 |
| Potassium carbonate |  |  | 5.00 |
| Premix^2^ | 2.30 | 2.30 | 2.30 |

^1^ Concentrates complemented the diets based on early (Fibre-) or late (Fibre+CP; Fibre+) harvested grass silage

^2^ The premix contained (per g): 8 mg Cu as CuSO_4_ 5H_2_O, 65 mg Zn as ZnO, 1 mg I as Ca(IO_3_)_2_, 31 mg Mn as MnO, 0.165 mg Co as CoSO_4_ 7H_2_O, 0.33 mg of Na_2_SeO_4_ 5H_2_O, 10000 IU of vitamin A, 800 IU vitamin D_3_ and 65 IU of vitamin E.

Supplementary Table S2 Nutrient composition of early or late harvested grass silages and of concentrates fed to dairy cows

|  |  | Grass silages | |  | Concentrates^1^ | | |
| --- | --- | --- | --- | --- | --- | --- | --- |
| Nutrients, g/kg DM |  | Early | Late |  | Fibre- | Fibre+CP | Fibre+ |
| DM (g/kg) |  | 407 | 465 |  | 887 | 908 | 897 |
| Crude protein |  | 238 | 118 |  | 106 | 564 | 241 |
| Crude fibre |  | 180 | 327 |  | 28.7 | 22.4 | 25.1 |
| NDF |  | 341 | 572 |  | 130 | 87 | 132 |
| ADF |  | 195 | 351 |  | 43.2 | 39.4 | 49.3 |
| Crude Fat |  | 54.4 | 27.3 |  | 35.4 | 64.4 | 33.2 |
| Ash |  | 93.8 | 80.5 |  | 56.2 | 86.7 | 85.6 |
| Mg |  | 1.63 | 1.47 |  | 4.96 | 5.70 | 6.14 |
| Ca |  | 6.79 | 4.89 |  | 6.01 | 13.4 | 14.1 |
| P |  | 4.00 | 3.46 |  | 3.14 | 7.08 | 6.49 |
| K |  | 33.8 | 30.2 |  | 4.72 | 10.7 | 7.60 |
| Na |  | 0.36 | 0.34 |  | 6.76 | 6.17 | 6.40 |
| NE_L ,_MJ |  | 7.1 | 5.2 |  | 8.1 | 7.3 | 7.5 |
| PDIE |  | 92 | 73 |  | 96 | 220 | 175 |
| PDIN |  | 149 | 74 |  | 70 | 358 | 171 |

^1^ Concentrates complemented the diets based on early (Fibre-) or late (Fibre+CP; Fibre+) harvested grass silage

NEL = Net energy for lactation. Protein digestible in the small intestine (Vérité *et al.*, 1979) calculated from its non-degradable N and degradable N contents (PDIN) or its rumen available energy content (PDIE)
